# Phase II trial designs for complex interventions: a single-arm single-stage design for clustered continuous outcomes

**DOI:** 10.1186/1745-6215-16-S2-P234

**Published:** 2015-11-16

**Authors:** Duncan T Wilson, Rebecca EA Walwyn, Sarah R Brown, Julia Brown, Amanda J Farrin

**Affiliations:** 1 Leeds Institute of Clinical Trials Research, Leeds, UK

## 

To incorporate formal assessments of potential efficacy into feasibility and pilot studies of complex interventions, appropriate methods for sample size determination are required. In particular, the implications of clustered patient outcomes resulting from cluster randomisation or treatment provision must be addressed. In this paper we will show how small-sample methods used to design phase II drug trials can be extended to allow for this clustering. In particular, we will propose a single-arm single-stage design for continuous data clustered in a two-level hierarchical structure. A number of alternative methods for determining the choice of sample size will be described. We will report the results of a simulation study comparing these methods in terms of the resulting error rates, suggested sample size parameters, and computational burden. A sensitivity analysis will demonstrate the implications of nuisance parameter misspecification at the design stage. Particular attention will be given to the scenario where the number of cluster-level units is severely limited, as will often be the case in the early phases of complex intervention development and evaluation.

